# Niosomes of active *Fumaria officinalis* phytochemicals: antidiabetic, antineuropathic, anti-inflammatory, and possible mechanisms of action

**DOI:** 10.1186/s13020-020-00321-1

**Published:** 2020-05-01

**Authors:** Karim M. Raafat, Sally A. El-Zahaby

**Affiliations:** 1 Department of Pharmaceutical Sciences, Faculty of Pharmacy, Beirut Arab University, Beirut, 115020 Lebanon; 2 Department of Pharmaceutics and Pharmaceutical Technology, Faculty of Pharmacy and Drug Manufacturing, Pharos University in Alexandria, Alexandria, Egypt

**Keywords:** *Fumaria officinalis*, Niosomes, HPLC-analysis, Antineuropathic, Anti-inflammatory

## Abstract

**Background:**

*Fumaria officinalis* (*F. officinalis,* FO) has been used in many inflammatory and painful-ailments. The main aim of this work is to perform an in-depth bio-guided phytochemical investigation of *F. officinalis* by identifying its main-active ingredients. Optimizing pharmacokinetics via niosomal-preparation will also be done to enhance their in vivo antineuropathic and anti-inflammatory potentials, and to explore their possible-mechanism of actions.

**Methods:**

Bio-guided phytochemical-investigations including fractionation, isolation, chromatographic-standardization, and identification of the most active compound(s) were done. Optimized niosomal formulations of *F. officinalis* most active compound(s) were prepared and characterized. An in vivo biological-evaluation was done exploring acute, subchronic, and chronic alloxan-induced diabetes and diabetic-neuropathy, and carrageenan-induced acute inflammatory-pain and chronic-inflammatory edema.

**Results:**

In-vivo bio-guided fractionation and chromatographic phytochemical-analysis showed that the alkaloid-rich fraction (ARF) is the most-active fraction. ARF contained two major alkaloids; Stylopine 48.3%, and Sanguinarine 51.6%. In-vitro optimization, analytical, and in vivo biological-investigations showed that the optimized-niosome, Nio-2, was the most optimized niosomal formulation. Nio-2 had particle size 96.56 ± 1.87 nm and worked by improving the pharmacokinetic-properties of ARF developing adequate entrapment-efficiency, rapid-degradation, and acceptable stability in simulated GI conditions. FO, ARF, and Nio 2 were the most potent antidiabetic and anti-inflammatory compounds. The reduction of the pro-inflammatory tumor necrosis factor-alpha (TNF-alpha) and Interleukin 6 (IL-6), and elevation the anti-inflammatory factor IL-10 levels and amelioration of the in vivo oxidative-stress might be the main-mechanism responsible for their antinociceptive and anti-inflammatory activities.

**Conclusions:**

*Fumaria officinalis* most-active fraction was identified as ARF. This study offers an efficient and novel practical oral formulation ameliorating various inflammatory conditions and diabetic complications especially neuropathic-pain.
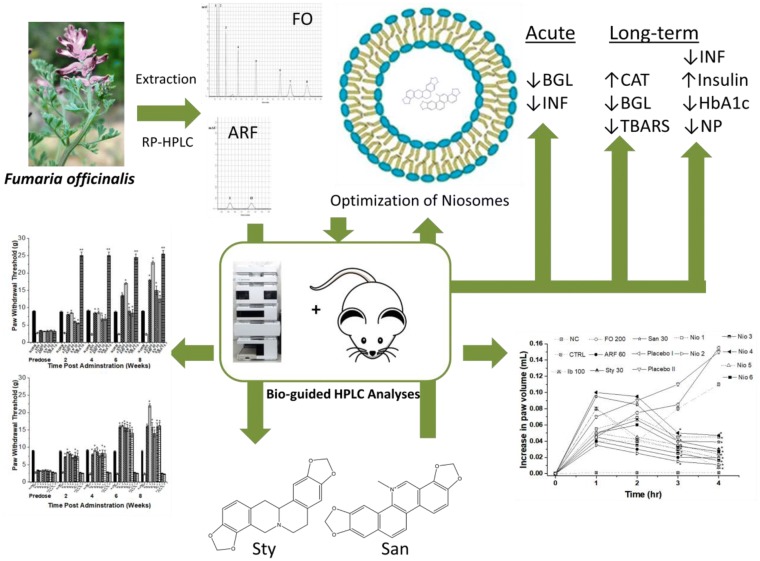

## Background

During the last decade, there was a growing demand for natural plants having diverse activities towards diseases especially chronic ones that need long term management [1]. *Fumaria officinalis*, family *Papaveraceae (Fumariaceae)*, also named “smoke of the earth” is a tiny plant that grows in many Eastern-Mediterranean countries. It has been used in the Asian folk-medicine in many inflammatory and painful ailments like conjunctivitis and rheumatism [2–5]. Additionally, researchers had proven its efficacy as an antioxidant, antiviral and antimicrobial agent [6]. The plant phytochemically comprises many secondary metabolites especially the isoquinoline alkaloids [7–9]. These alkaloids are determined in literature by diverse techniques mainly reversed Phase-HPLC methods [8]. The folk use of *F. officinalis* (*Fumaria officinalis*) in various chronic ailments, made it of interest to explore its effect on other inflammatory and metabolic disorders and their complications.

Diabetes mellitus is a chronic metabolic-syndrome distinguished by the blood glucose level elevation, which in-turn increases the oxidative stress leading to many complications like diabetic neuropathy [10, 11]. Diabetic neuropathy is peripheral micro-vascular neuropathy in which sensory and motor nerves are affected leading to the progressive loss of sensation and is characterized by hyperalgesia and allodynia. Besides metabolic factors, ischemic factors and inflammation also contribute to the development of diabetic neuropathies. The oxidative stress can lead to activation of the inflammatory mediators like cytokinins which contributes to nerve hypoxia [12, 13].

Inflammation is a defensive mechanism against pathogenic-aggression or noxious-stimuli. Interleukin (IL)-6, TNF-alpha (tumor necrosis factor-alpha), and elevated oxidative stress play an important part in the acute painful inflammatory pathogenesis and its chronic complications like edema [14, 15].

Despite, the biological efficiency of some natural drugs, the application of pharmaceutical formulations might have a good impact on the overall activity of these drugs because of the improvement in pharmacokinetics. One of these formulations is the utilization of niosomes. L’Oreal developed and patented niosomes since the 1970s for cosmetic purposes [16]. Then researchers successfully developed plenty of niosomal drug delivery formulations in different disciplines [17–19]. Niosomes are microscopic vesicles made of nonionic surfactant and cholesterol that are formed upon hydration in aqueous media [20]. Niosomes are supremacy compared to liposomes mainly in terms of stability which originated from their nonionic surfactant composition. Moreover, niosomes are more economical, more easily prepared, and devoid of undesired solvents usage [20]. These vesicles can be thermodynamically stable if the formulator chooses the right surfactant mixtures and the proper stabilizer, which is usually cholesterol. Additionally, the temperature at which the vesicles form must be adjusted above the gel/liquid transition of the main lipid composition of niosomes [21]. Niosomes are nanoscopic vesicles that can hold both hydrophilic and lipophilic drugs and are used as either targeted carriers for drugs, hormones, and antigens to diverse body organs or to control the release of these agents [22].

The literature survey has shown that there are some in vitro preliminary experiments done on the anti-diabetic activity of *F. officinalis* [5, 23], but there is no comprehensive work on optimizing its pharmacokinetics, in vivo antineuropathic potentials and the phytochemicals responsible for its activity and their mechanisms of action.

Thus, the objective of this work is to perform an in-depth bio-guided phytochemical investigation of *F. officinalis* identifying its main-active ingredients. Optimizing pharmacokinetics via niosomal-preparation will also be done to enhance their in vivo antineuropathic and anti-inflammatory potentials, and exploring their possible-mechanism of actions.

## Materials and methods

### Plant collection, extraction, and phytochemical standardization

*Fumaria officinalis* (Papaveraceae) aerial parts (*F. officinalis*) were collected from Akkar district, Lebanon (N 34° 33′ 08″ E 36° 11′ 49″, Lat. 34.5521507 Lng. 36.1970030) on March 2019. The herb was authenticated by comparing it to a reference sample, and a representative sample was stored in the faculty herbarium under the voucher specimen number (PS-38-18) for future reference.

*Fumaria officinalis* was dried in shade and was extracted utilizing Centic sonicator (China) using ethanol (80%) for 120 min in three rounds. The extract was then dried under vacuum utilizing the Buchi rotary-evaporator (Germany). The obtained dry extract was sonicated with 0.05 M H_2_SO_4_ then filtered. Furthermore, the filtrate was treated with 30% NH_4_OH, EtAc, and re-dried via rotary-evaporator. The dried extract was kept under − 40 °C until further utilization.

The extract was standardized using Agilent HPLC (Japan) apparatus. An RP-HPLC method was utilized comprising a degasser, a C-18 column, and a mobile phase consisted of ACN (40%) and Triethylamine (60%) and the flow rate was adjusted to 1 mL/min utilizing a DAD-detector focusing on 254 and 280 nm. The HPLC peaks were identified and quantified by comparing to standard calibration curves of reference standards, and standard steeping methods [24]. The main peaks were confirmed utilizing in-line fraction separator. Each fraction was then injected in Nano-ESI mass spectrometer to affirm the main active peaks. All standards and solvents (analytical-grade) were purchased from Merck (Germany).

### Bio-guided fractionation, isolation, and identification of the most active compound(s)/fraction

To find *F. officinalis* most active compound(s)/fraction, *F. officinalis* was fractionated utilizing a column chromatography method. The glass column (500 mm × 3000 mm) utilized RP-silica gel as a stationary phase. A gradient mobile phase was utilized using a mixture of (A) acetonitrile and (B) triethylamine (0.1%); starting with 100% B, then 40% A, and finally 100% A. Two-hundred fractions were gathered, and each fraction was investigated for its hypoglycemic and antinociceptive effects. The most active compound(s)/fraction were identified utilizing a similar RP-HPLC method used for the standardization of the whole extract. Mass spectrometry, ^1^H and ^13^C NMR-spectroscopy of the most bioactive fraction was also measured in deuterated DMSO on a Bruker 300 NMR-spectrometer at room temperature. The most active compound(s)/fraction were used to formulate the niosomes formulations.

### Niosomes materials, preparation, and characterization

Span 60 (S) and Brij 52 (B) were purchased from Merck (Germany). Cholesterol (CH, purity 98%) was obtained from Amresco (USA). All other chemicals (analytical grade) were obtained from Merck (Germany).

Various niosomal-formulations were prepared by ether-injection method [25]. In brief, in ether-injection, Span 60 or Brij 52 and CH were dissolved in ether. This solution was gradually injected into warm phosphate-buffer saline (pH = 7.4, 65 °C) comprising the most active fraction and rotating (100 rpm) at a constant rate. Niosomal vesicles were formed after the removal of ether and stored in a refrigerator (Table 1).

**Table 1:** The composition and characteristics of the tested niosomal formulations

Code	Composition^a^	Weight ratio	EE (%)	Zeta potential (mV)	Z-average (nm)	PDI^b^
Nio1	S:CH	1:1	91.25 ± 2.80	− 53.06 ± 2.40	90.60 ± 1.90	0.30 ± 0.01
Nio2	S:CH	2:1	94.65 ± 3.33	− 56.75 ± 2.60	96.56 ± 1.87	0.34 ± 0.02
Nio3	S:CH	3:1	92.56 ± 4.21	− 58.10 ± 3.10	97.60 ± 1.55	0.36 ± 0.01
Nio4	S:B	1:1	96.70 ± 2.30	− 67.80 ± 3.40	93.56 ± 2.22	0.31 ± 0.01
Nio5	S:B	2:1	98.22 ± 1.44	− 65.38 ± 3.20	94.66 ± 2.34	0.33 ± 0.02
Nio6	S:B	3:1	93.55 ± 1.99	− 58.50 ± 3.00	98.7 ± 1.22	0.36 ± 0.01
Placebo I	S:CH	3:1	–	− 25.70 ± 2.45	86.66 ± 1.11	0.28 ± 0.02
Placebo II	S:B	3:1	–	− 24.60 ± 2.68	84.99 ± 1.33	0.27 ± 0.01

^a^*S* Span 60, *CH* cholesterol, *B* Brij 52

^b^*PDI* poly-dispersity index

Entrapment efficiency, vesicle-size, zeta potential, and in vitro profile release of the various niosomes were detected.

### Entrapment efficiency (EE)

To determine the content of the *F. officinalis* (FO) most active fraction in the niosomal formulations, the un-entrapped content was separated from the niosomes utilizing the membrane-dialysis method against buffer solution at 4 °C [25]. The vesicles were washed using phosphate buffered saline followed by 1 h centrifugation. The amount of entrapped FO was determined by lysis of the vesicles with absolute ethanol. The concentration of FO most active fraction in the resulting solution was measured utilizing RP-HPLC. The % EE was determined using the following equation:

$$\% {\text{ EE}}\, = \,{\text{Amount of entrapped FO most active fraction }} \times \, 100/{\text{Total amount}}$$

### Vesicle size and zeta potential

The vesicle size and zeta potential of various niosomal formulations were investigated utilizing Malvern Zeta-sizer (UK) at ambient temperature [25]. After repeating the experiments three times, Z-average (nm) and zeta potential (mV) values were given as the mean ± mean standard error (SEM).

### In vitro drug release

The release of FO most active fraction from niosomal formulations was studied using membrane-dialysis. Half a milliliter of FO most active fraction-loaded niosomes was placed into cellulose membrane-dialysis which were then transferred to 50 mL of the release media (pH = 6.8, enzyme-free simulated intestinal fluid (SIF) containing Tween 80 (0.1%, w/v), to maintain the sink condition, and sodium taurocholate (10 mM), to simulate bile-salt concentration in the small-intestine, as reported before in the literature [25]. To mimic the release system, the system was maintained at 37 ± 0.5 °C was magneti-cally stirred at 100 rpm. Aliquots of release media (1 mL) were removed, and replaced with fresh media, on a fixed intervals (0, 0.25, 0.5, 1.0, 1.5, and 3 h), centrifuged, and examined utilizing the RP-HPLC method. This method was repeated three times and the average release was determined (mean ± SEM).

### Niosomes stability in sodium taurocholate

Under perfect sink conditions, the ability of FO active fraction-loaded niosomes to keep their physical-properties was tested when accompanied by sodium taurocholate (STC, 10 mM). Various niosomes were placed into SIF (37 °C) containing STC (10 mM) and stirred (110 rpm). Placebo I (drug-free Nio 3) and Placebo II (drug-free Nio 6) were prepared and were investigated the same way as the FO active fraction-loaded niosomes.

Vesicular size, zeta potential, and poly-dispersity index (PDI) were measured prior to and directly after 10 h incubation utilizing Malvern Zeta-sizer (UK). The results were given as an average (mean ± SEM) of three experimental replications.

### Animals and in vivo experiments

Male Swiss albino mice (20–28 g) were used in the in vivo experiments. Animals had free entry to the water. Mice had free access to standard laboratory food. As for diabetes experiments, the mice were made to fast for 16 h. Animals were exposed to 12 h dark–light cycles. The protocol of the experimental design (n = 7/group) is summarized in (Table 2). Animal care for the research was done abiding by BAU Institutional Review Board regulations (2019A-0051-P-R-0341).

**Table 2:** Protocol of experimental design

Groups	n	Tested substance(s)	Description
A. Acute (6 h) and subchronic (8 days) effect of *Fumaria officinalis* (FO) on blood glucose levels			
I	7	Control	Normal mice: Vehicle [sterile cold saline (0.9%)], PO
II	7	Diabetic Control	Diabetic mice: Vehicle, PO
III	7	GB	Diabetic mice: GB 5 mg/kg, PO
IV	7	*FO*	Diabetic mice: *FO* 50 mg/kg, PO
V	7	*FO*	Diabetic mice: *FO* 100 mg/kg, PO
VI	7	*FO*	Diabetic mice: *FO* 200 mg/kg, PO
VII	7	ARF	Diabetic mice: ARF 15 mg/kg, PO
VIII	7	ARF	Diabetic mice: ARF 30 mg/kg, PO
IX	7	ARF	Diabetic mice: ARF 60 mg/kg, PO
X	7	Placebo I	Diabetic mice: Placebo I (Nio 3), PO
XI	7	Nio 1	Diabetic mice: ARF 60 mg/kg in Nio 1, PO
XII	7	Nio 2	Diabetic mice: ARF 60 mg/kg in Nio 2, PO
XIII	7	Nio 3	Diabetic mice: ARF 60 mg/kg in Nio 3, PO
XIV	7	Placebo II	Diabetic mice: Placebo II (Nio 6), PO
XV	7	Nio 4	Diabetic mice: ARF 60 mg/kg in Nio 4, PO
XVI	7	Nio 5	Diabetic mice: ARF 60 mg/kg in Nio 5, PO
XVII	7	Nio 6	Diabetic mice: ARF 60 mg/kg in Nio 6, PO
XVIII	7	Sty	Diabetic mice: Sty 7.5 mg/kg, PO
XIX	7	Sty	Diabetic mice: Sty 15 mg/kg, PO
XX	7	Sty	Diabetic mice: Sty 30 mg/kg, PO
XXI	7	San	Diabetic mice: San 7.5 mg/kg, PO
XXII	7	San	Diabetic mice: San 15 mg/kg, PO
XXIII	7	San	Diabetic mice: San 30 mg/kg, PO
B. For longer time (0, 2, 4, 6 and 8 weeks) effects on hot plate and tail withdrawal latencies, and von Frey paw withdrawal thresholds			
XXIII	7	Control	Normal mice: Vehicle [sterile cold saline (0.9%)], PO
XXIV	7	Vehicle Control	Diabetic mice: Vehicle, PO
XXV	7	GB	Diabetic mice: GB 5 mg/kg, PO
XXVI	7	*FO*	Diabetic mice: *FO* 50 mg/kg, PO
XXVII	7	*FO*	Diabetic mice: *FO* 100 mg/kg, PO
XXVIII	7	*FO*	Diabetic mice: *FO* 200 mg/kg, PO
XXIX	7	ARF	Diabetic mice: ARF 15 mg/kg, PO
XXX	7	ARF	Diabetic mice: ARF 30 mg/kg, PO
XXXI	7	ARF	Diabetic mice: ARF 60 mg/kg, PO
XXXII	7	Placebo I	Diabetic mice: Placebo I (Nio 3), PO
XXXIII	7	Nio 1	Diabetic mice: ARF 60 mg/kg in Nio 1, PO
XXXIV	7	Nio 2	Diabetic mice: ARF 60 mg/kg in Nio 2, PO
XXXV	7	Nio 3	Diabetic mice: ARF 60 mg/kg in Nio 3, PO
XXXVI	7	Placebo II	Diabetic mice: Placebo II (Nio 6), PO
XXXVII	7	Nio 4	Diabetic mice: ARF 60 mg/kg in Nio 4, PO
XXXVIII	7	Nio 5	Diabetic mice: ARF 60 mg/kg in Nio 5, PO
XXXIX	7	Nio 6	Diabetic mice: ARF 60 mg/kg in Nio 6, PO
XXXX	7	Sty	Diabetic mice: Sty 7.5 mg/kg, PO
XXXXI	7	Sty	Diabetic mice: Sty 15 mg/kg, PO
XXXXII	7	Sty	Diabetic mice: Sty 30 mg/kg, PO
XXXXIII	7	San	Diabetic mice: San 7.5 mg/kg, PO
XXXXIV	7	San	Diabetic mice: San 15 mg/kg, PO
XXXXXV	7	San	Diabetic mice: San 30 mg/kg, PO

### Diabetes and diabetic-neuropathy biological evaluation

Diabetes experiments were performed for 6 h (acute), 8 days (subchronic), and 8 weeks (chronic). The acute and the subchronic experiments were performed utilizing Sigma gluco-stripes and glucometers (Germany). The chronic studies were done utilizing Analyticon HbA1c mini-columns (Germany). The diabetes was provoked in mice by alloxan injection (180 mg/Kg) for 3 days. Mice having blood glucose levels (BGL) ≥ 200 mg/Kg and HbA1c levels ≥ 8% were considered diabetic and were incorporated in the in vivo experiments [26].

Diabetic neuropathy was confirmed after 8 weeks of provoking diabetes with paw withdrawal thresholds less than 5 g [27]. The diabetic neuropathy (DN) symptoms of hyperalgesia were evaluated (in seconds) utilizing UgoBasile hot plate device (Italy) and Hugo-Sachs-Elektronik tail flick device (Germany) with a cut-off of 10S. DN symptoms of mechanical allodynia were measured (in grams) using Opti-hair Von Frey filaments (Germany) [28].

### Anti-diabetic and antinociceptive mechanism of action

To understand the anti-diabetic and antinociceptive mechanism of action attributed to *F. officinalis* and its active phytochemicals, serum insulin level (SIL) was evaluated pre-administration and 8 weeks post-dosing. SIL was recorded prior-to and 8 weeks post-test administration using an Agilent HPLC device (Japan) and utilizing Merck reversed phase-C18 (Germany) with a one ml/min flow-rate, and column temperature adjusted to 40 °C at 214 nm. The gradient mobile phase was composed of acetonitrile (A) and 0.1% trifluoroacetic acid in Milli-Q water (B) and launched from 0 to 5 min 30% (A) and then from 5 to 15 min 40% (A) [29]. The inhibitory effects of the tested compounds on alpha-glucosidase inhibitory and alpha-amylase inhibitory potentials were determined in accordance with the methods described before [27]. Moreover, the elevated BGL increases the oxidative stress marked by the decrease of serum catalase levels (CAT), reduced glutathione levels (GSH), and elevate lipid peroxidase levels (LPO). This increase in oxidative stress was reported to be responsible for diabetes comorbidities like painful DN [30, 31]. Thus, the evaluation of antioxidant CAT, GSH, and LPO might give us insight into the *F. officinalis* antinociceptive mechanism of action. Furthermore, CAT levels were evaluated (kU/l) by a modified method described before [32]. Also, change in GSH levels were measured (µg/mg) at predose and 8 weeks post-oral-administration [33]. Also, LPO was evaluated utilizing TBARS (Thiobarbituric acid reactive substances) levels (nM/100 g) utilizing a modified method described previously using JASCO-spectrophotometer (Japan) at 532 nm [34].

### Anti-inflammatory biological evaluation

The anti-inflammatory activities of *F. officinalis* and its active phytochemicals/formulas were evaluated acutely via the carrageenan-induced inflammatory-pain method, and chronically via hind-paw edema method [35].

Acutely, the Merck carrageenan-solution (100 μL, 1% in saline, Germany) was injected into the left hind-paw intra-plantarly. The positive control, Ibuprofen 100 mg/Kg (Ib), was orally dispensed 30.00 ± 1.00 min before carrageenan-injection [36, 37]. The vehicle control mice (VEH) administered saline (100 μL) only prior to carrageenan-injection. The evaluation of behavior was done 2 h after carrageen-injection.

The hind-paw edema method was performed chronically utilizing a modified method described before [35]. In brief, after the oral administration of the active phytochemicals/formulas, the volume of the carrageenan-injected paw edema was determined utilizing MRPP plethysmograph (China) after administration and 4 h afterward, utilizing a positive control, Ib 100 mg/Kg.

### The anti-inflammatory mechanism of action

To understand the anti-inflammatory mechanism of action attributed to *F. officinalis* and its active phytochemicals, the level of inflammatory-mediators was determined. After centrifugation of the tissue-homogenates, the cytokines were measured in the supernatant using TNF-alpha, IL-6, and IL-10 (Bio-Legend, USA) enzyme-linked immune-sorbent assay (ELISA) kits [38].

### Statistical analysis

Statistical analyses were done with OriginPro (USA). Data are shown as the mean ± SEM. Comparison of the pharmacokinetic-parameters and the formulations were performed utilizing analysis-of-variance. In vivo experiments were statistically evaluated by one way ANOVA followed up with the Student–Newman–Keuls analysis. *P* < 0.05 was regarded as statistically-significant.

## Results

### Bio-guided phytochemical investigation

**Fig. 1:**
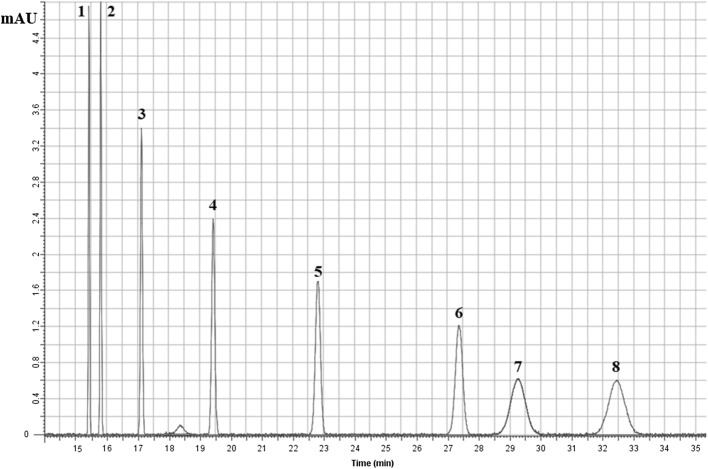
*Fumaria officinalis* RP-HPLC major peaks: (1) Cheleritrine (9.2%), (2) Hydrastine (10.7%), (3) Bicuculline (11.2%), (4) Protopine (12.3%), (5) Chelidonine (13.2%), (6) Allocryptopine (13.8%), (7) Stylopine (14.3%), and (8) Sanguinarine (15.3%). The mobile phase consisted of ACN (40%) and Triethylamine (60%) and the flow rate was 1 mL/min utilizing DAD detector focusing on 254 and 280 nm

Utilizing the RP-HPLC method, the *F. Officinalis* extract (FO) was standardized. The FO HPLC chromatogram has shown that it contains 8 major peaks: (1) Cheleritrine (9.2%) (2) Hydrastine (10.7%) (3) Bicuculline (11.2%) (4) Protopine (12.3%), (5) Chelidonine (13.2%), (6) Allocryptopine (13.8%), (7) Stylopine (14.3%), and (8) Sanguinarine (15.3%) (Fig. 1).

**Fig. 2:**
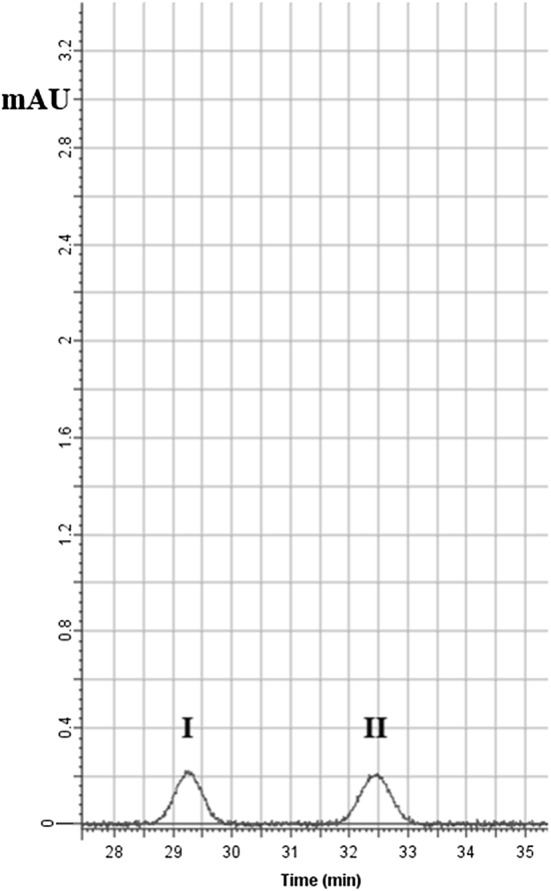
RP-HPLC of the alkaloid rich fraction (ARF) major peaks: (I) Stylopine (48.3%), and (II) Sanguinarine (51.6%). The mobile phase consisted of ACN (40%) and Triethylamine (60%) and the flow rate was 1 mL/min utilizing DAD detector focusing on 254 and 280 nm

**Fig. 3:**
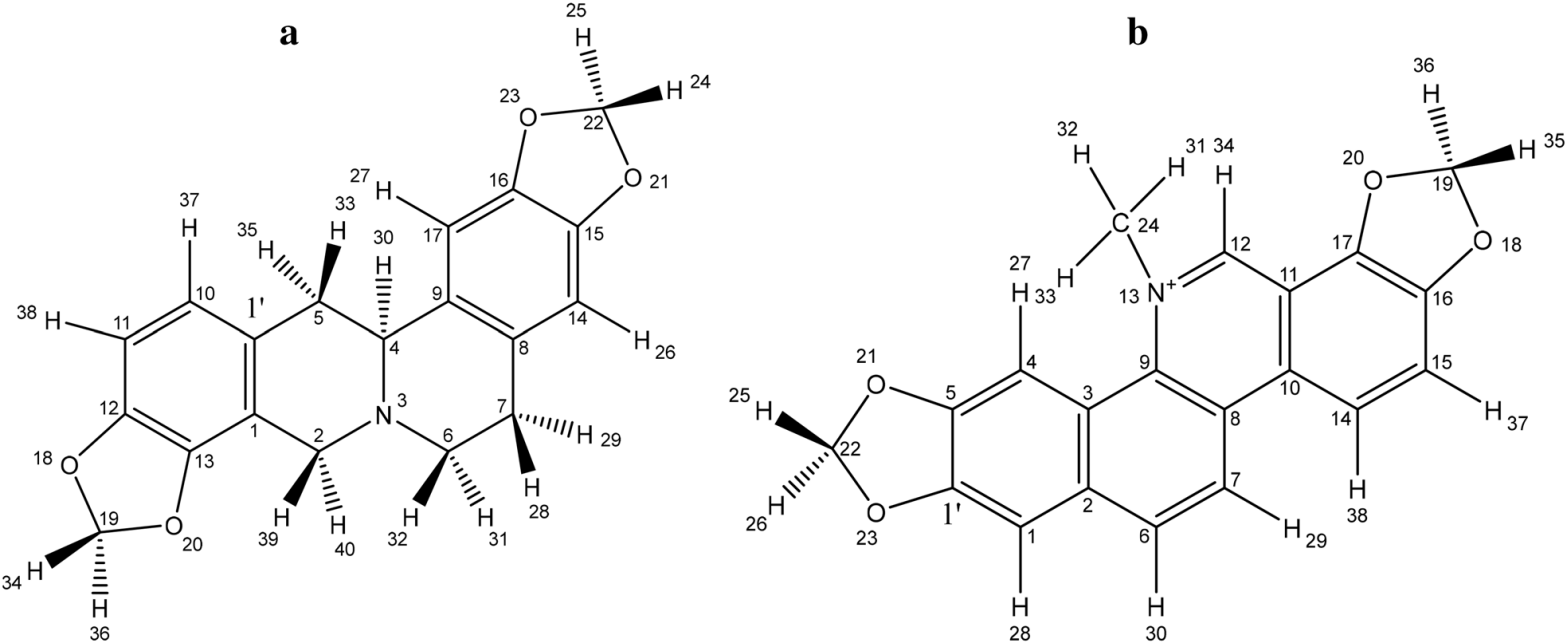
The alkaloid rich fraction (ARF) major constituents’ chemical structures: **a** Stylopine and **b** Sanguinarine

A preliminary in vivo bio-guided phytochemical screening assay was performed on about 200 fractions isolated from FO. The bio-guided phytochemical screening assay has shown that the most active fraction, possessing comparable in vivo results to that of FO, is rich in alkaloids, hence named the alkaloid-rich fraction (ARF). The RP-HPLC investigation utilizing standard steeping and calibration curves method has shown that ARF contains 2 major peaks: Stylopine (Sty, 48.3%), and (II) Sanguinarine (San, 51.6%) (Figs. 2 and 3). Moreover, fractionation of ARF has been performed using flash chromatography and Sty and San were isolated and identified chromatographically. These findings were confirmed utilizing in-line fraction separator and positive Nano-ESI–MS/MS system (Mwt: Sty, 324.3 g/mol, and San, 333.09 g/mol). ^1^H and ^13^C NMR have confirmed that Sty and San are the most active compounds in ARF (Table 3).

**Table 3:** ^1^H -NMR and ^13^C- NMR data

Structure	Position	δC	Position	δH, m, (*J* in Hz)
Stylopine^a^	1	120.8	24	5.93, d (*J *= 10.62 Hz)
	1′	133.2	25	5.92, d (*J *= 10.61 Hz)
	2	53.7	26	6.54, d (*J *= 0.44 Hz)
	3	–	27	6.29, d (*J *= 0.43 Hz)
	4	58.9	28	2.72, ddd (*J *= 13.46, 10.18, 3.41 Hz)
	5	36.1	29	2.83, ddd (*J *= 13.46, 3.41, 2.23 Hz)
	6	51.2	30	4.03, dd (*J *= 10.15, 3.49 Hz)
	7	28.5	31	2.69, ddd (*J *= 13.96, 10.18, 3.14 Hz)
	8	127.3	32	3.04, ddd (*J *= 13.96, 3.41, 2.23 Hz)
	9	129.8	33	2.76, dd (*J *= 13.34, 3.49 Hz)
	10	126.3	34	5.85, d (*J *= 10.51 Hz)
	11	108.1	35	2.78, dd (*J *= 13.33, 10.15 Hz)
	12	145.3	36	5.92, d (*J *= 10.52 Hz)
	13	142.7	37	6.42, d (*J *= 8.62 Hz)
	14	108.0	38	6.37, d (*J *= 8.61 Hz)
	15	147.3	39	3.89, d (*J *= 11.90 Hz)
	16	145.1	40	3.87, d (*J *= 11.91 Hz)
	17	109.5		
	18	–		
	19	101.4		
	20	–		
	21	–		
	22	101.2		
	23	–		
Sanguinarine^a^	1	106.3	25	33.32, d (*J *= 15.50 Hz)
	1′	148.7	26	33.33, d (*J *= 15.50 Hz)
	2	132.9	27	7.65, t (*J *= 0.44 Hz)
	3	128.2	28	7.64, dt (*J *= 1.55, 0.44 Hz)
	4	106.3	29	8.10, dt (*J *= 6.19, 0.43 Hz)
	5	148.0	30	8.12, ddd (*J *= 6.19, 1.55, 0.46 Hz)
	6	126.7	31	4.19, s
	7	126.5	32	4.18, s
	8	128.5	33	4.19, s
	9	138.4	34	9.71, d (*J *= 0.55 Hz)
	10	130.0	35	6.51, d (*J *= 15.50 Hz)
	11	116.7	36	6.50, d (*J *= 15.51 Hz)
	12	144.2	37	7.03, d (*J *= 8.81 Hz)
	13	–	38	8.53, ddd (*J *= 8.83, 0.55, 0.44 Hz)
	14	126.1		
	15	114.8		
	16	147.4		
	17	142.5		
	18	–		
	19	101.1		
	20	–		
	21	–		
	22	101.3		
	23	–		
	24	46.1		

^a^Figure 2

### Niosomes optimization

Preliminary studies for niosome formulations were performed using different types of surfactants, surfactants ratio and preparation methodology. Cholesterol is reported to help acquiring homogeneity for the niosomal dispersions so it is included in all the prepared formulations [39]. Composition and method of preparation of niosomes were then chosen based on the obtained in vitro release pattern and the physicochemical properties of FO most active fraction, ARF. A series of formulations were prepared, alter-ing both surfactants and Cholesterol (CH) content while preserving the other factors unchanged. The best surfactants that enhanced ARF in vitro release pattern were span 60 and Brij 52. The best method of niosome preparation was found to be the ether injection method that enabled the highest entrapment efficiency (EE). Thus, six ARF preparations (Nio 1–6) were prepared utilizing CH and either span 60 or Brij 52byether injection method. As shown in Table 3, all niosomes yielded high EE in the range from 91.25 ± 2.80 to 98.22 ± 1.44. The size of niosomal preparations ranged from 84.99 ± 1.33 to 98.7 ± 1.22 nm with PDI in the range of 0.27 ± 0.01 to 0.36 ± 0.01, which is accepted and indicated homogeneity of the prepared formulations [40]. Comparing Nio 1 with Nio 4, it is obvious that Span 60 yielded smaller vesicles size than Brij 52, this can be explained based on the fact that surfactants having longer alkyl chains usually form larger niosomal vesicles [41].

Zeta-potential measurements were − 24.60 ± 2.68 to − 67.80 ± 3.40 mV indicating very good stability [42]. The observed negative charge was owing to hydroxyl-ions adsorption on the niosomal surface as previously reported [25, 43].

### The in vitro drug release from niosomal formulations

**Fig. 4:**
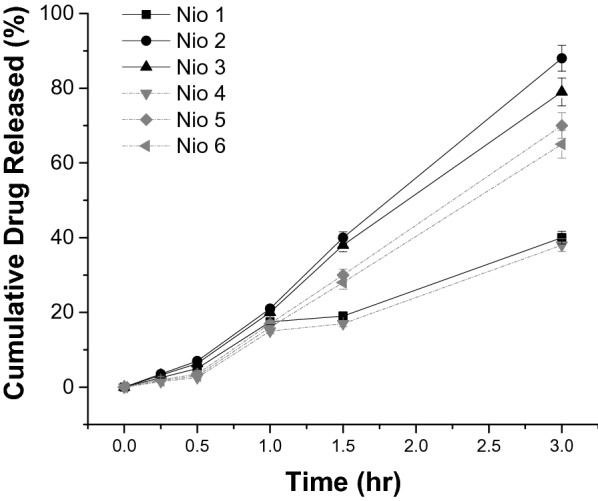
In-vitro release pattern of alkaloid rich fraction (ARF) from different niosomes (mean ± SEM)

In-vitro release study was performed to identify the influence of formulations optimization on the drug release rate from the niosomal-carriers. The cumulative release of ARF from Nio 1–6 were illustrated in Fig. 4. The cumula-tive drug release was approximately: Nio 1 (40.01 ± 1.72%), Nio 2 (88.12 ± 3.43%), Nio 3 (79.22 ± 3.71%), Nio 4 (38.24 ± 1.69%), Nio 5 (69.98 ± 3.39%), and Nio 6 (64.88 ± 3.79%), within 3 h (Fig. 4). It is obvious that increasing Span 60 (Nio 2 and 3) had increased the release of ARF compared to Nio 1. This could be attributed to the solubilizing power of Span 60 which facilitated the drug dispersion into the release media.

### Niosomes stability in sodium taurocholate

The ability of ARF-loaded niosomes to retain their physical properties in the presence of sodium taurocholate under perfect sink conditions was investigated. Nio 1–6 were positioned into a pre-warmed release medium (enzyme-free) containing sodium taurocholate (10 mM) and was stirred (110 rpm) at 37 °C.

**Fig. 5:**
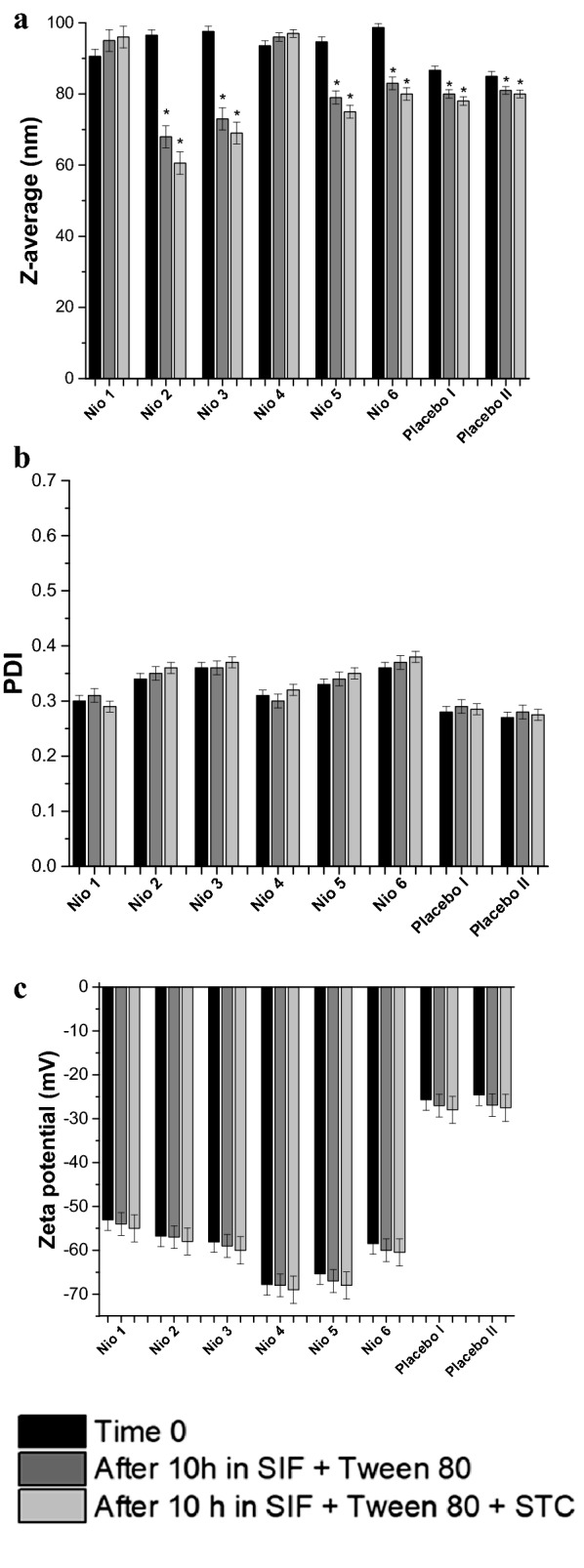
Different niosomal formulations before and after 10 h incubation at 37 °C in SIF with bile salts: **a** Z-average (nm), **b** PDI, and **c** Zeta potential (mV). Data represent the mean ± SEM (n = 3). Where SIF is the simulated intestinal fluid and STC is sodium taurocholate. *P value < 0.05 versus time 0

Vesicular size distributions, zeta potential, and poly-dispersity index (PDI) were measured prior to and directly after 10 h incubation utilizing Malvern Zeta-sizer (UK). The results of the influence of bile salts on the stability of ARF-loaded niosomes after incubation are described in Fig. 5. As shown in Fig. 5a, significant decreases (*P* < 0.05) in particle size (Z-average) were observed in Nio 2, Nio 3, Nio 5, Nio 6, and placebo 1 and II. Tween 80 and STC diffusion through dialysis membrane might enable them to reach the niosomal formulations affecting their physicochemical-characteristics, as described before in the literature [25]. On the other hand, there were no significant differences in z-average in Nio 1 and Nio 4, which indicated stability of all formulations after incubation in STC. Furthermore, after 10 h incubation, no significant changes of PDI in Tween 80 alone or with STC were observed that ensured stability of the vesicles and that they preserved their acceptable range for oral delivery (Fig. 5b). Zeta-potentials are shown in Fig. 5c, where, no significant changes were observed in their values. This means that optimization of the prepared niosomal formulations had succeeded in their protection against harsh gastrointestinal conditions. This conclusion was based on the collective measurements of particle size, Zeta potential and PDI, where, the particle size was still within the acceptable range, Zeta potential values were sufficiently high and PDI values indicated homogenous distribution of the formed niosomes. The results were also within the allowed ranges for oral delivery, as described previously with similar dispersions [25].

### In-vivo diabetes and diabetic-neuropathy biological evaluation

**Fig. 6:**
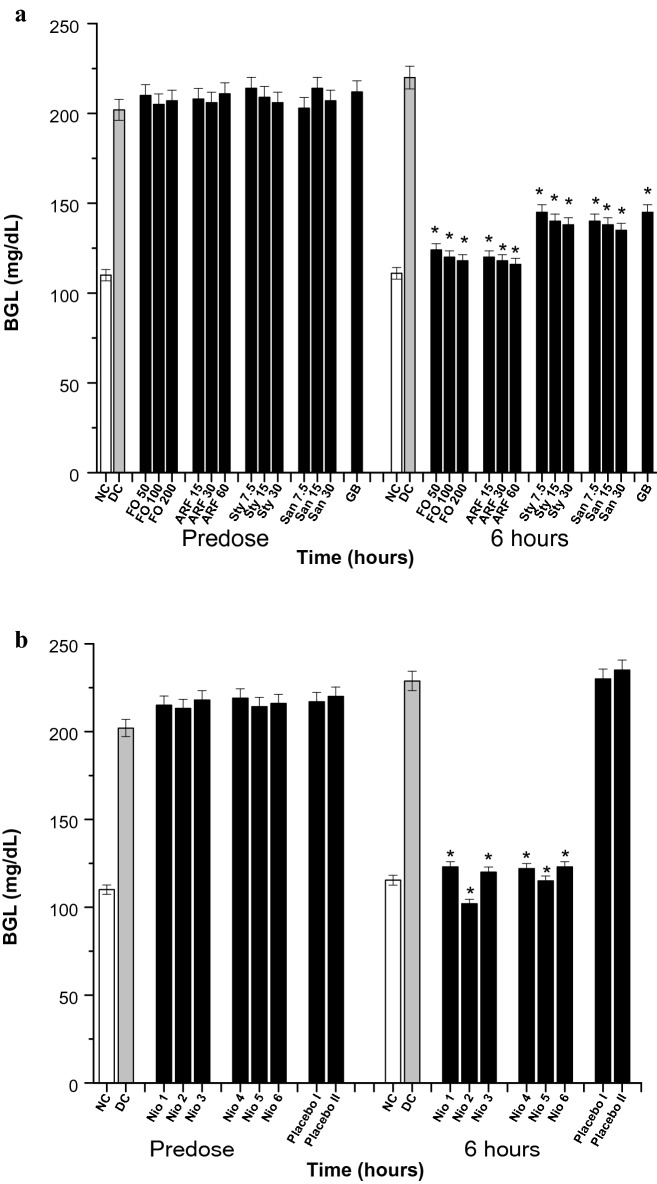
Acute BGL. All doses are in (mg/Kg). **a** The acute effects of FO, ARF, Sty and San various doses utilizing Glibenclamide 5 mg/Kg (GB) as a positive control. **b** The acute effects of Nio 1–6 and placebo I and II. Asterisks designate significant results (p < 0.05) when compared to diabetic control (DC). NC designates normal non-diabetic control

**Fig. 7:**
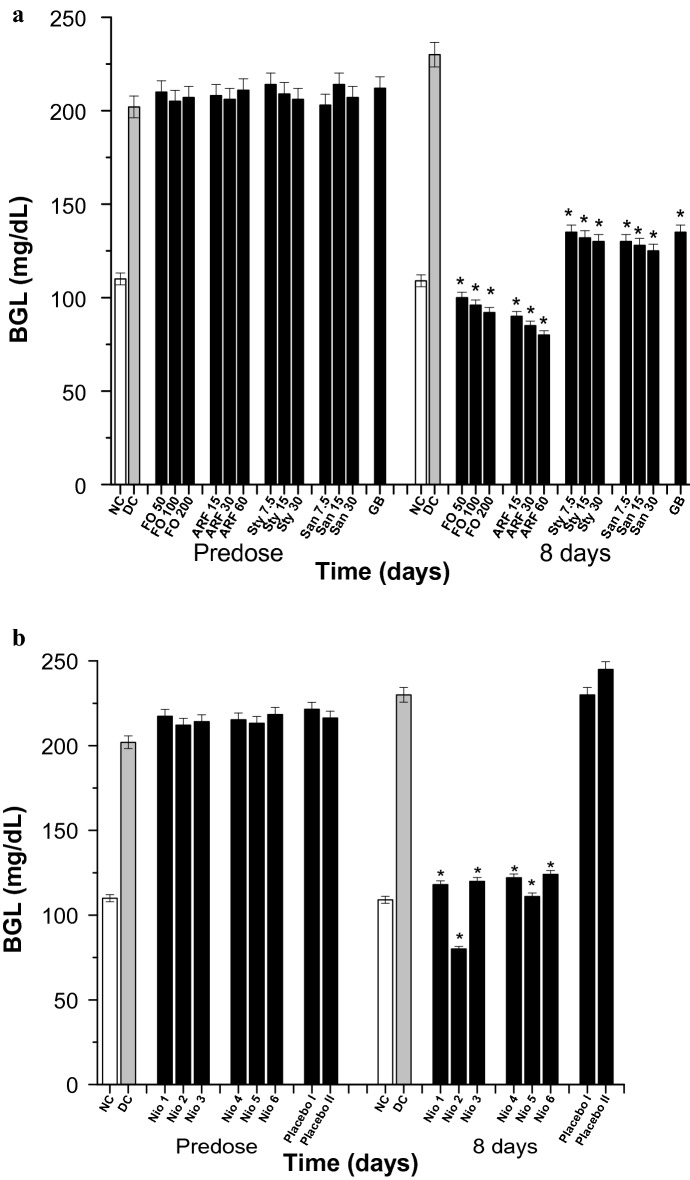
Subchronic BGL. All doses are in (mg/Kg). **a** The subchronic effects of FO, ARF, Sty and San various doses utilizing Glibenclamide 5 mg/Kg (GB) as a positive control. **b** The subchronic effects of Nio 1–6 and placebo I and II. Asterisks designate significant results (p < 0.05) when compared to diabetic control (DC). NC designates normal non-diabetic control

**Fig. 8:**
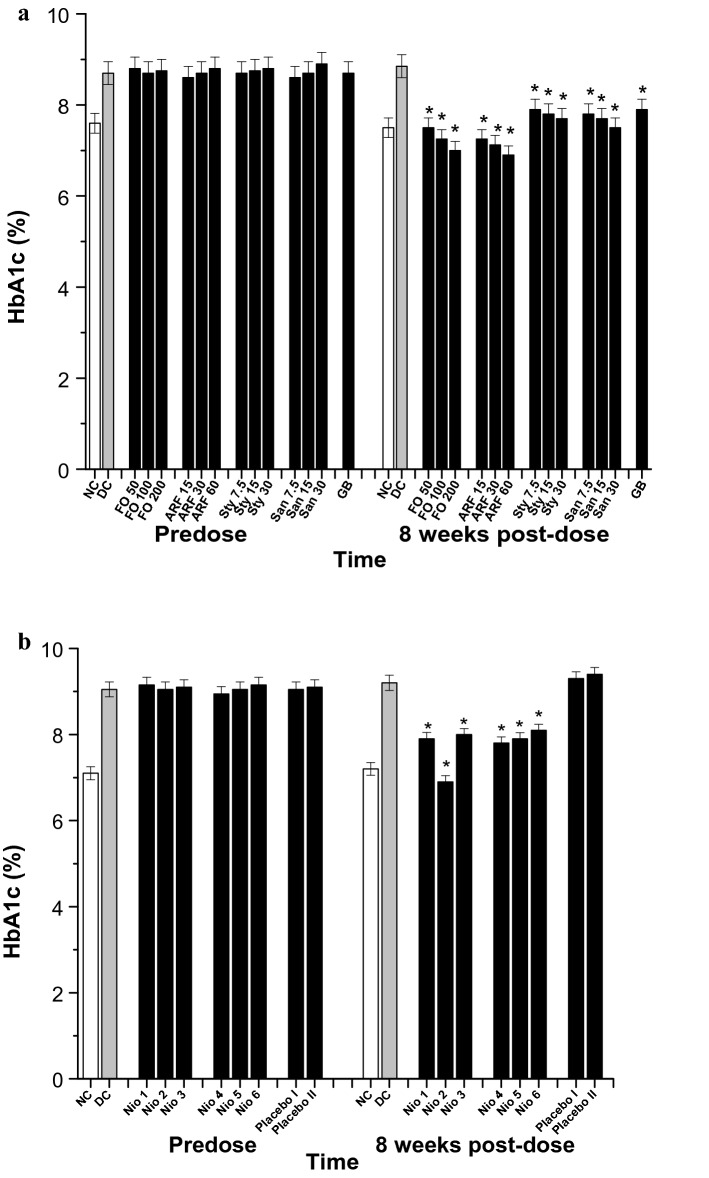
HbA1c (%) analysis. All doses are in (mg/Kg). **a** The effects of FO, ARF, Sty and San various doses utilizing Glibenclamide 5 mg/Kg (GB) as a positive control. **b** The effects of Nio 1–6 and placebo I and II. Asterisks designate significant results (p < 0.05) when compared to diabetic control (DC). NC designates normal non-diabetic control

The anti-diabetic potentials of the tested compounds were evaluated acutely for 6 h (Fig. 6), subchronically for 8 days (Fig. 7) utilizing Sigma glucometers (Germany), and chronically for 8 weeks (Fig. 8) using Analyticon HbA1c mini-columns (Germany).

Acutely, *Fumaria officinalis* (FO) has shown a significant (*P* ˂ 0.05) and dose dependent hypoglycemic activity, when compared to vehicle control diabetic mice (DC). After 6 h of oral administration, FO (50, 100, and 200 mg/Kg) have shown 43.64, 45.45, and 46.36% reduction in blood glucose level (BGL), respectively (Fig. 6a). Moreover, FO most active fraction, ARF, has shown comparatively more significant (*P* < 0.05) BGL reduction than FO. After 6 h of oral administration, ARF (15, 30, and 60 mg/Kg) have shown 45.54, 46.63, and 47.27% BGL reduction, respectively (Fig. 6a). Furthermore, ARF components stylopine (Sty), and sanguinarine (San) have been tested the same way as ARF. After 6 h of oral administration, Sty (7.5, 15, and 30 mg/Kg) have shown 34.10, 36.64, and 37.72% BGL reduction, respectively, while that of San (7.5, 15, and 30 mg/Kg) have shown 36.36, 37.27, and 38.63% BGL reduction, respectively (Fig. 6a). These results indicate that ARF, acutely, has the most superior antidiabetic activity when compared to FO, ARF individual components (Sty and San), and the positive control, Glibenclamide (GB) (Fig. 6a). Thus, to observe the optimization effects of various niosomal formulations (Nio 1–6), ARF was utilized to be the active ingredient in these dispersions and was tested the same way as ARF. The doses of Nio 1–6 were adjusted to contain equivalent amounts of the highest dose of ARF (200 mg/Kg). Placebo I (Nio 3 deprived of ARF) and placebo II (Nio 6 deprived of ARF) niosomes were also tested, as a negative control (Fig. 6b). After 6 h of oral administration, Nio 1, Nio 2, Nio 3, Nio 4, Nio 5, and Nio 6 have shown 46.24, 55.42, 47.55, 46.68, 49.74, and 46.42% BGL reduction, respectively (Fig. 6b). Placebo I and II did not show any significant hypoglycemic activity, implying the efficiency of these formulations and that the formula improved the pharmacokinetics without having any anti-diabetic effects (Fig. 6b).

Subchronically, FO has shown a significant (*P* < 0.05) and dose dependent hypoglycemic activity, when compared to DC. As after 8 days of oral administration, FO (50, 100, and 200 mg/Kg) have shown 56.52, 58.26, and 60.10% reduction in blood glucose level (BGL), respectively (Fig. 7a). Additionally, ARF has shown comparatively more significant (*P* < 0.05) BGL reduction than FO. As after 8 days of oral administration, ARF (15, 30, and 60 mg/Kg) have shown 60.87, 63.04, and 65.21% BGL reduction, respectively (Fig. 7a). In addition, Sty (7.5, 15, and 30 mg/Kg) have shown 41.30, 42.61, and 43.48% BGL reduction, respectively, whilst that of San (7.5, 15, and 30 mg/Kg) have shown 43.47, 44.35, and 45.65% BGL reduction, respectively (Fig. 7a). These results strengthen the acute findings that ARF has the most superior antidiabetic activity when compared to FO, Sty, San, and GB (Fig. 7a). Furthermore, Nio-1, Nio-2, Nio-3, Nio-4, Nio-5, and Nio-6 have shown 48.70, 65.22, 47.82, 46.96, 51.74, and 46.09% BGL reduction, respectively (Fig. 7b). Placebo I and II also did not show any significant hypoglycemic activity, implying the efficiency of these formulations and that the formula improved the pharmacokinetics without having any anti-diabetic effects (Fig. 7b).

Chronically, the effect of the tested compounds was monitored for their changes in HbA1c (%) levels prior to and 8 weeks after test compounds’ administration in the diabetic groups (Fig. 8). FO has shown a significant (*P* < 0.05) and dose dependent normalization of HbA1c levels when compared to DC (Fig. 8a). After 8 weeks of FO administration, FO (50, 100, and 200 mg/Kg) have shown 15.25, 18.08, and 20.90% HbA1c level reduction (Fig. 8a). Additionally, ARF has shown comparatively more significant (*P* < 0.05) HbA1c level reduction than FO. As after 8 weeks of oral administration, ARF (15, 30, and 60 mg/Kg) have shown 18.09, 19.49, and 22.03% HbA1c level reduction, respectively (Fig. 8a). Furthermore, Sty (7.5, 15, and 30 mg/Kg) have shown 10.73, 11.86, and 13.00% HbA1c level reduction, respectively, whilst that of San (7.5, 15, and 30 mg/Kg) have shown 11.86, 12.98, and 15.25% HbA1c level reduction, respectively (Fig. 8a). These results strengthen the previous findings that ARF has the most superior antidiabetic activity when compared to FO, Sty, San, and GB (Fig. 8a). Moreover, Nio-1, Nio-2, Nio-3, Nio-4, Nio-5, and Nio-6 have shown ca. 10.73, 22.03, 9.60, 11.86, 10.73, and 8.47% HbA1c level reduction, respectively (Fig. 8b). As expected, placebo I and II did not show any significant HbA1c level changes (Fig. 8b).

### Anti-diabetic mechanism of action

**Fig. 9:**
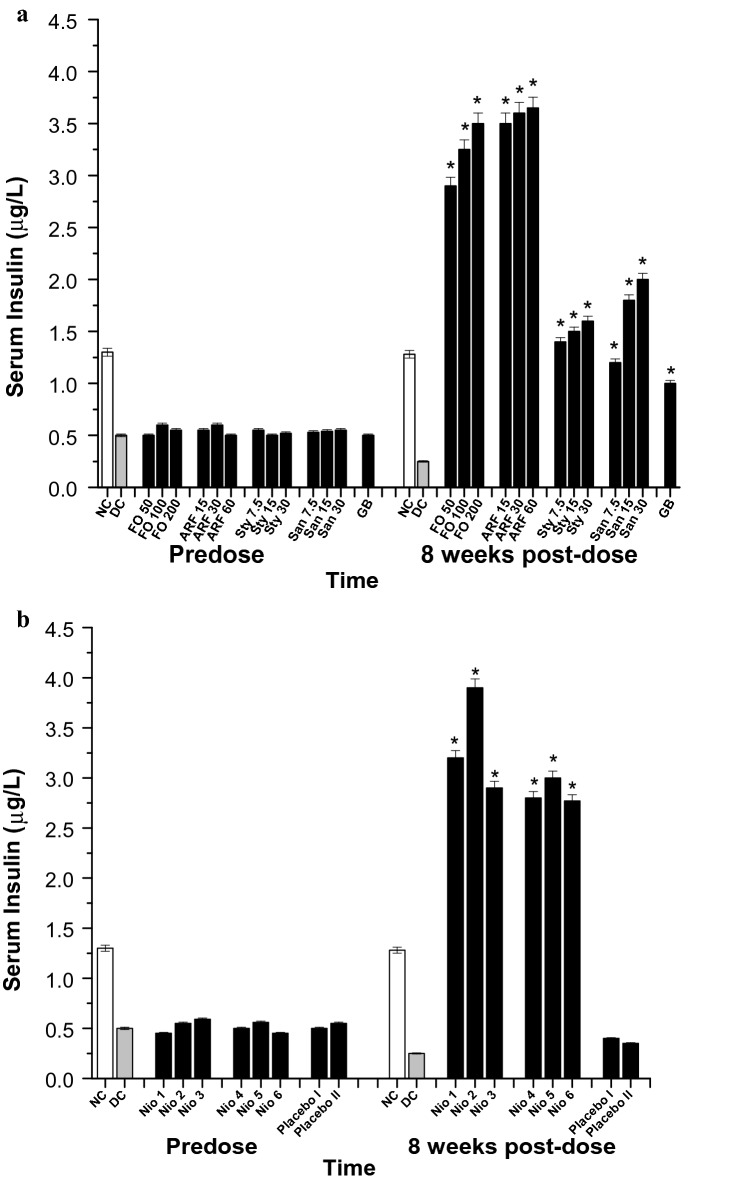
Serum insulin (µg/L). All doses are in (mg/Kg). **a** The effects of FO, ARF, Sty and San various doses utilizing Glibenclamide 5 mg/Kg (GB) as a positive control. **b** The effects of Nio 1–6 and placebo I and II. Asterisks designate significant results (p < 0.05) when compared to diabetic control (DC). NC designates normal non-diabetic control

In order to explore the mechanism of actions underlying the tested compounds anti-diabetic activity, the serum insulin levels (SIL) of the various diabetic groups were monitored prior to and 8 weeks post-test compounds administration (Fig. 9). After 8 weeks of FO administration, FO (50, 100, and 200 mg/Kg) have shown 106, 120, and 130 fold increase in SIL (Fig. 9a). Furthermore, ARF (15, 30, and 60 mg/Kg) have shown ca. 131, 134, and 136 fold increase in SIL, respectively (Fig. 9a). Additionally, Sty (7.5, 15, and 30 mg/Kg) have shown ca. 46, 50, and 54 fold increase in SIL, respectively, whilst that of San (7.5, 15, and 30 mg/Kg) have shown ca. 38, 62, and 70 fold increase in SIL, respectively (Fig. 9a). These results imply that FO and ARF have potential antidiabetic activities and that their insulin-secretagogue potentials might be responsible for their hypoglycemic activities. Both FO and ARF have shown a dose dependant alpha-amylase and alpha-glucosidase inhibitory effects (Table 2). Also, Nio-1, Nio-2, Nio-3, Nio-4, Nio-5, and Nio-6 have shown ca. 118, 146, 106, 102, 110, and 101 fold increases in SIL, respectively (Fig. 9b). Placebo I and II did not show any significant SIL changes (Fig. 9b).

### Diabetic-neuropathy biological evaluation

**Fig. 10:**
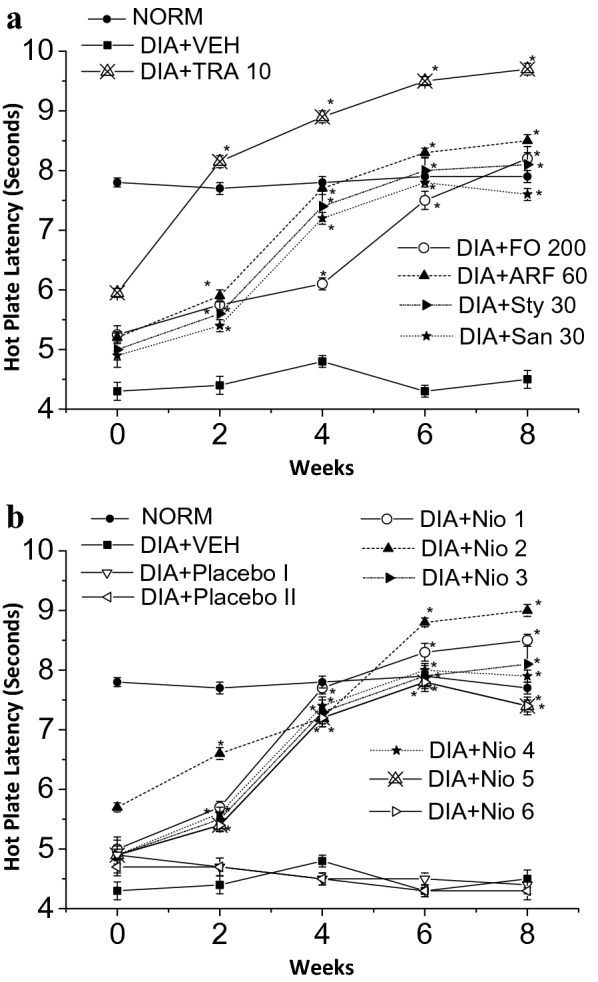
Thermal hyperalgesia hot plate latency test. All doses are in (mg/Kg). **a** The effects of FO, ARF, Sty and San highest doses utilizing Tramadol 10 mg/Kg (TRA 10) as a positive control. **b** The effects of Nio 1–6 and placebo I and II. Asterisks designate significant results (p < 0.05) when compared to diabetic control (DC). NC designates normal non-diabetic control

**Fig. 11:**
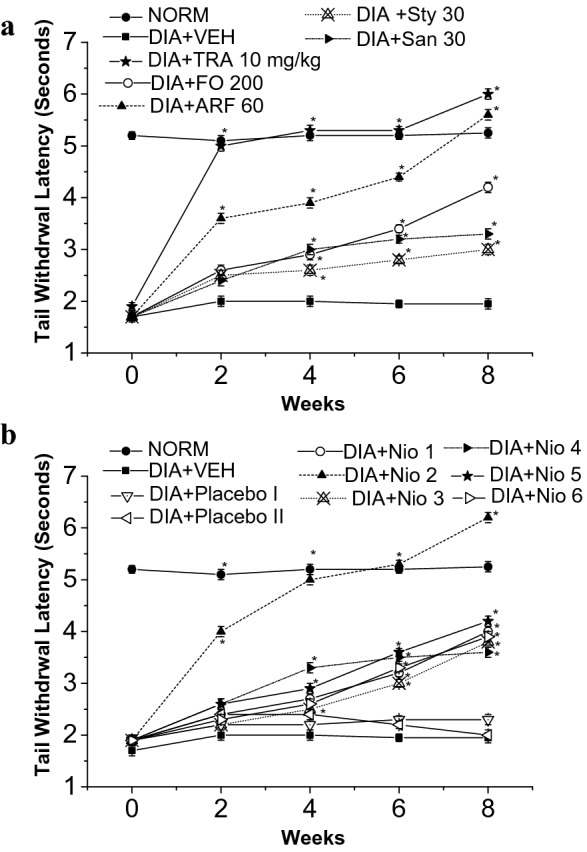
Thermal hyperalgesia tail withdrawal latency test. All doses are in (mg/Kg). **a** The effects of FO, ARF, Sty and San highest doses utilizing Tramadol 10 mg/Kg (TRA 10) as a positive control. **b** The effects of Nio 1–6 and placebo I and II. Asterisks designate significant results (p < 0.05) when compared to diabetic control (DC). NC designates normal non-diabetic control

**Fig. 12:**
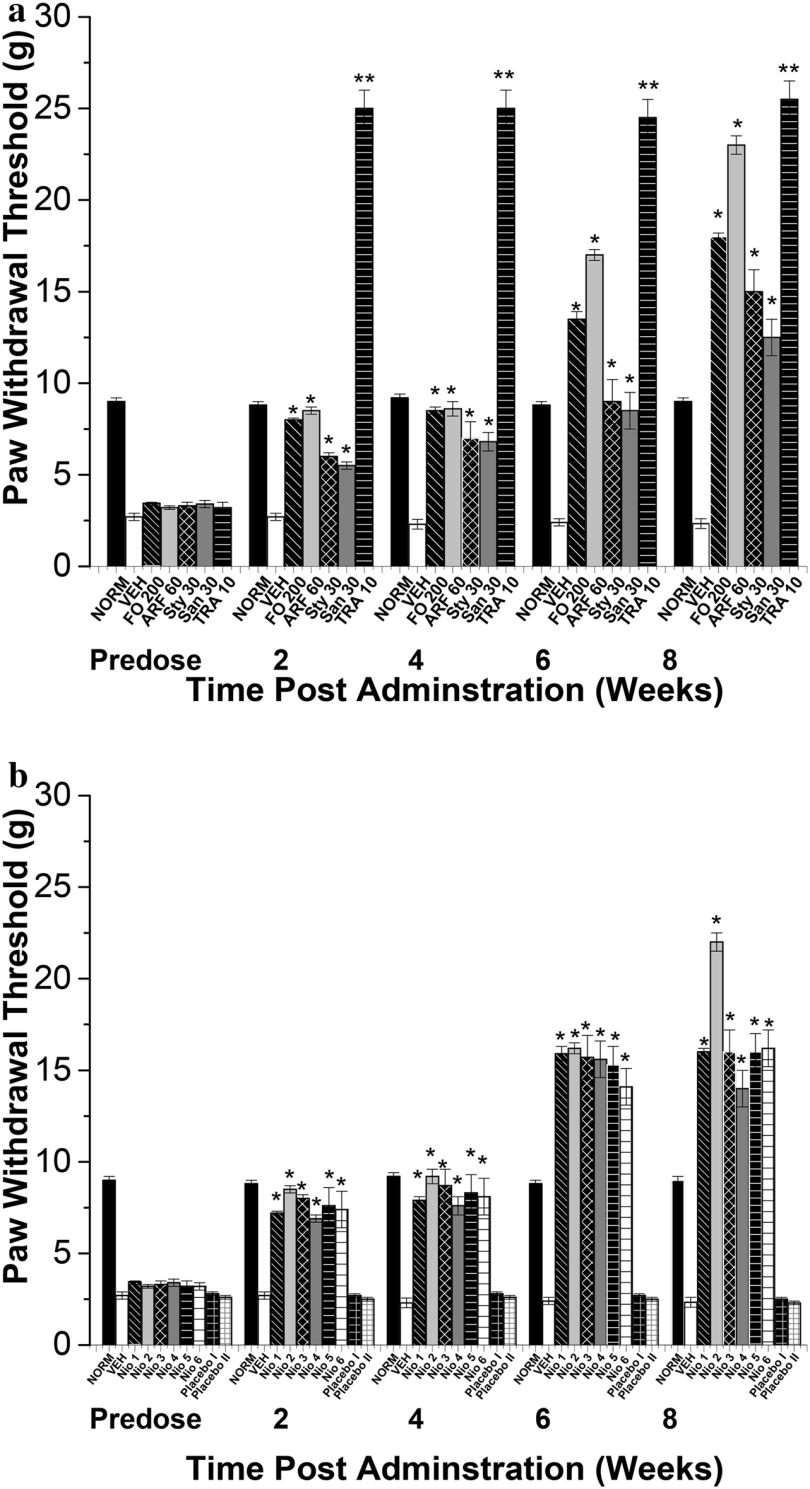
Mechanical allodynia Von Frey test. All doses are in (mg/Kg). **a** The effects of FO, ARF, Sty and San highest doses utilizing Tramadol 10 mg/Kg (TRA 10) as a positive control. **b** The effects of Nio 1–6 and placebo I and II. Asterisks designate significant results (p < 0.05) when compared to diabetic control (DC). NC designates normal non-diabetic control

After 8 weeks of stimulation of diabetes, the diabetic animals were tested for symptoms of diabetic neuropathy. Diabetic animals with paw withdrawal thresholds less than 5 g were considered neuropathic and were included in the experiments [27]. The diabetic neuropathy symptoms of hyperalgesia (assessed by hot-plate and tail flick experiments) (Figs. 10 and 11) and allodynia (assessed by Von Frey filaments) (Fig. 12) were monitored chronically for 8 weeks post diabetic neuropathy symptoms provoking. Tramadol 10 mg/Kg (TRA) was utilized as a positive control. The tested compounds were given every other day for 8 weeks to assess their effects on symptoms of diabetic neuropathy (Figs. 10, 11, 12).

When compared to vehicle-treated group (VEH), the highest doses of FO (200 mg/Kg), ARF (60 mg/Kg), Sty (30 mg/Kg), and San (30 mg/Kg) have shown 82.22, 88.89, 79.99, 68.89%, respectively, improvement in hot-plate latency (Fig. 10a), and ca. 11.5, 18.7, 6.9, 5.4 fold respectively, improvement in tail-flick latency (Fig. 11a), 8 weeks post-administration. These results showed that FO and ARF had potential anti-hyperalgesic potentials, as shown previously with similar compounds [44]. Moreover, Nio-1, Nio-2, Nio-3, Nio-4, Nio-5, and Nio-6 have shown ca. 88.89, 99.98, 79.98, 75.56, 73.33, and 71.11%, respectively, improvement in hot-plate latency (Fig. 10b), and ca. 1.2, 2.2, 1.0, 0.8, 1.1, and 0.9 fold, respectively, improvement in tail-flick latency (Fig. 11b), 8 weeks post-administration. Placebo I and II did not show any significant changes in hot-plate and tail-flick latency experiments.

Compared to VEH, FO (200 mg/Kg), ARF (60 mg/Kg), Sty (30 mg/Kg), and San (30 mg/Kg) highest doses have shown ca. 67.3, 88.7, 54.4, 43.6 fold, respectively, improvement in paw withdrawal thresholds (PWT) utilizing Von Frey filaments (Fig. 12), 8 weeks post-administration. These results showed that FO and ARF have significantly (*P* < 0.05) alleviated the mechanical allodynia provoked by hyperglycemia, as demonstrated previously with analogous compounds [24]. Furthermore, Nio-1, Nio-2, Nio-3, Nio-4, Nio-5, and Nio-6 have shown ca. 58.7, 84.4, 58.6, 49.9, 58.6, and 59.5 fold, respectively, improvement in PWT (Fig. 12b), 8 weeks post-administration. Placebo I and II did not show any significant changes in PWT experiments (Table 4).

**Table 4:** Alpha-glucosidase and alpha-amylase inhibitory effects

Group	Dose (mg/kg)	Alpha-amylase inhibition (%)	Alpha-glucosidase inhibition (%)
FO	50	32.18 ± 1.75^a^	49.12 ± 1.05 ^g^
FO	100	49.73 ± 1.70^b^	51.83 ± 1.00 ^h^
FO	200	63.78 ± 1.31^c^	71.48 ± 1.01^i^
ARF	15	33.15 ± 1.46^d^	54.44 ± 1.06^j^
ARF	30	53.63 ± 1.56^e^	56.36 ± 1.03 ^k^
ARF	60	69.23 ± 1.43^f^	74.33 ± 1.07 ^l^

Values are shown as mean ± S.E.M. (n = 3).Values with different letters are significantly different (p ≤ 0.05)

*S.E.M.* mean standard error

### Antinociceptive mechanism of action

To explore the mechanism of actions underlying the tested compounds anti-diabetic neuropathy activities, the levels of the in vivo antioxidants (CAT, GSH, and LPO) serum levels were monitored for various diabetic-neuropathy groups prior to and 8 weeks post test compounds administration (Table 5). After 8 weeks of FO administration, FO (50, 100, and 200 mg/Kg) have shown 79.11, 90.71, and 104.33% increase in CAT levels, respectively (Table 5). Furthermore, ARF (15, 30, and 60 mg/Kg) have shown ca. 94.99, 99.59, and 120.23% increase in CAT levels, respectively, ca. 85.28, 87.50, 88.89% reduction in TBARS level, respectively, and ca. 37.36, 39.35, and 47.74% elevation in GSH levels, respectively (Table 5). In addition, Sty (7.5, 15, and 30 mg/Kg) have shown 53.88, 63.98, and 69.78% increase in CAT levels, respectively, ca. 73.05, 73.61, 74.17% reduction in TBARS level, respectively, and ca. 24.35, 25.52, and 25.96% elevation in GSH levels, respectively, whilst that of San (7.5, 15, and 30 mg/Kg) have shown 59.03, 59.38, and 65.99% increase in CAT levels, respectively, ca. 74.17, 75.28, 76.39% reduction in TBARS level, respectively, and ca. 26.19, 26.26, and 28.75% elevation in GSH levels, respectively (Table 5). These results imply that FO and ARF have potential antioxidant activities combating the oxidative stress provoking the diabetic neuropathy [11]. Furthermore, these data have shown that FO and ARF anti-oxidative stress potentials and insulin-secretagogue long-term anti-diabetic activities might be responsible for their antidiabetic neuropathy potentials, as seen with similar natural products [45].

**Table 5:** In vivo assessment of the antioxidant activities of *Fumaria officinalis* on CAT levels in serum, alterations in TBARS and reduced GSH (Mean ± S.E.M., n = 7/group)

Group	Dose (mg/kg)	Catalase level (kU/I)		TBARS Level (nM/100 g)		GSH (µg/mg)	
		Predose	8th week	Predose	8th week	Predose	8th week
NC	–	30.77 ± 1.66	30.20 ± 1.44	0.73 ± 0.02	0.75 ± 0.02	64.40 ± 1.10	64.83 ± 1.50
DC	–	21.28 ± 1.20	19.82 ± 1.48	1.15 ± 0.02	3.60 ± 0.03	57.30 ± 1.70	47.72 ± 1.40
GB^a^	5	21.42 ± 1.11	22.17 ± 1.36	1.17 ± 0.02	1.78 ± 0.04	56.70 ± 1.60	56.53 ± 1.50
FO^a^	50	21.66 ± 1.44	35.50 ± 1.25*	0.98 ± 0.01	0.58 ± 0.02*	59.90 ± 1.50	64.9 ± 1.50*
FO^a^	100	21.98 ± 1.23	37.80 ± 1.43*	1.02 ± 0.01	0.56 ± 0.03*	59.00 ± 1.60	65.11 ± 1.30*
FO^a^	200	21.85 ± 1.55	40.50 ± 1.37*	1.00 ± 0.02	0.54 ± 0.04*	61.58 ± 1.30	65.50 ± 1.40*
ARF^a^	15	22.19 ± 1.15	38.65 ± 1.55*	0.97 ± 0.02	0.53 ± 0.01*	60.98 ± 1.40	65.55 ± 1.40*
ARF^a^	30	21.28 ± 1.78	39.56 ± 1.70*	1.00 ± 0.02	0.45 ± 0.01*	62.85 ± 1.20	66.50 ± 1.10*
ARF^a^	60	22.49 ± 1.57	43.65 ± 1.31*	0.96 ± 0.01	0.40 ± 0.02*	62.11 ± 1.30	70.50 ± 1.10*
Sty^a^	7.5	21.42 ± 1.25	30.50 ± 1.45*	1.15 ± 0.02	0.97 ± 0.03*	60.60 ± 1.20	59.34 ± 1.30*
Sty^a^	15	21.29 ± 1.11	32.50 ± 1.34*	1.02 ± 0.02	0.95 ± 0.03*	61.83 ± 1.50	59.90 ± 1.40*
Sty^a^	30	21.89 ± 1.45	33.65 ± 1.05*	0.97 ± 0.01	0.93 ± 0.01*	60.13 ± 1.70	60.11 ± 1.50*
San^a^	7.5	22.29 ± 1.15	31.52 ± 1.13*	1.00 ± 0.03	0.93 ± 0.03*	59.75 ± 1.60	60.22 ± 1.80*
San^a^	15	21.28 ± 1.18	31.59 ± 1.56*	1.02 ± 0.02	0.89 ± 0.02*	61.74 ± 1.20	60.25 ± 1.90*
San^a^	30	22.51 ± 1.16	32.90 ± 1.50*	0.98 ± 0.01	0.85 ± 0.02*	61.17 ± 1.50	61.44 ± 1.70*
Nio 1^a^	60	22.70 ± 1.78	37.56 ± 1.77*	0.92 ± 0.02	0.45 ± 0.02*	59.66 ± 1.40	63.22 ± 1.40*
Nio 2^a^	60	22.90 ± 1.34	46.25 ± 1.45*	1.22 ± 0.02	0.34 ± 0.02*	59.77 ± 1.21	75.55 ± 1.10*
Nio 3^a^	60	21.97 ± 1.56	39.56 ± 1.33*	0.89 ± 0.01	0.46 ± 0.02*	59.34 ± 1.33	65.66 ± 1.10*
Nio 4^a^	60	22.56 ± 1.77	33.58 ± 1.45*	0.94 ± 0.02	0.50 ± 0.01*	59.83 ± 1.54	62.99 ± 1.40*
Nio 5^a^	60	22.89 ± 1.44	35.65 ± 1.55*	1.01 ± 0.01	0.48 ± 0.02*	60.13 ± 1.70	65.55 ± 1.30*
Nio 6^a^	60	22.48 ± 1.35	34.60 ± 1.45*	0.90 ± 0.01	0.56 ± 0.01*	60.75 ± 1.55	63.88 ± 1.40*
Placebo I^a^	–	22.45 ± 1.33	20.22 ± 1.22	1.11 ± 0.01	1.30 ± 0.04	62.98 ± 1.40	60.00 ± 1.30*
Placebo II^a^	–	22.56 ± 1.77	19.20 ± 1.46	1.22 ± 0.02	1.45 ± 0.04	62.55 ± 1.41	59.33 ± 1.20*

*S.E.M.* mean standard error

* *p* < 0.05 significant from the vehicle control animals

^a^Compared to vehicle control

Moreover, Nio-1, Nio-2, Nio-3, Nio-4, Nio-5, and Nio-6 have shown ca. 89.51, 133.35, 99.59, 69.42, 79.87, and 74.57% increase in CAT serum levels, respectively, ca. 87.50, 90.56, 87.22, 86.11, 86.67, and 84.44% reduction in TBARS level, respectively, and ca. 32.48, 58.32, 37.59, 32.00, 37.36, and 33.86% elevation in GSH levels, respectively (Table 5). Placebo I and II did not show any significant CAT, TBARS, or GSH serum changes (Table 5).

### Anti-inflammatory biological evaluation

**Fig. 13:**
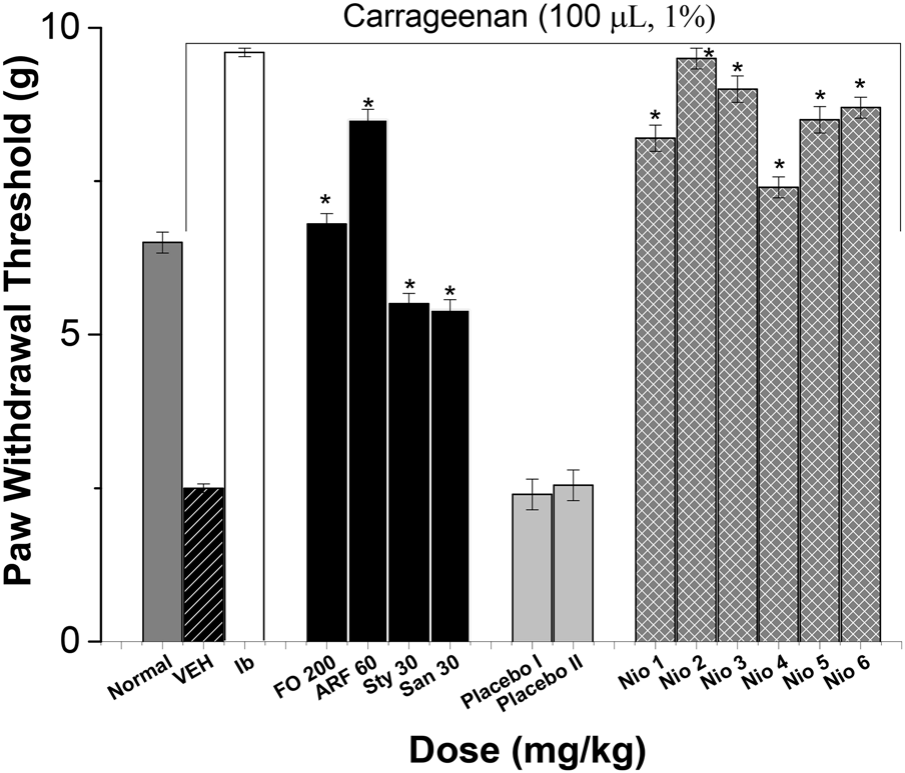
The effect on acute inflammatory pain. All doses are in (mg/Kg) utilizing Ibuprofen 100 mg/Kg (Ib) as a positive control. Asterisks designate significant results (p < 0.05) when compared to vehicle control (VEH). Normal designates normal non-carrageenan treated control

**Fig. 14:**
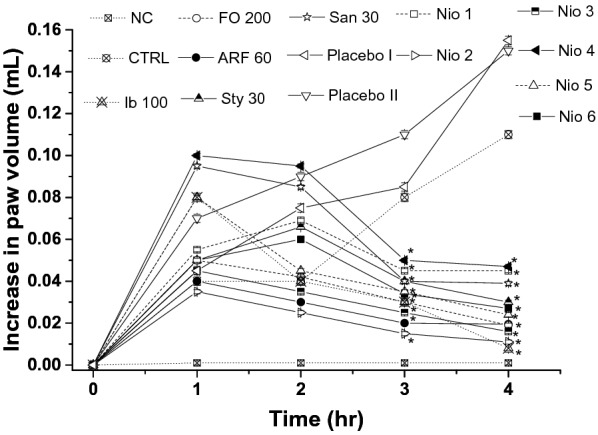
The anti-inflammatory effects on mouse hind paw edema. All doses were in mg/Kg. The actual edema volume increase was measured relative to that of standard drug ibuprofen 100 mg/kg. Values are presented as mean ± SEM,  = 7. “*” denotes significant difference from control value at  < 0.05

The anti-inflammatory potentials of FO, ARF, Sty, San, and various formulas were evaluated acutely via carrageenan-induced inflammatory-pain method (Fig. 13), and chronically via hind-paw edema method (Fig. 14), as previously done with analogous compounds [35] utilizing ibuprofen 100 mg/Kg (Ib) as a positive control (Figs. 13 and 14).

The acute carrageenan-induced inflammatory-pain method has shown that FO (200 mg/Kg), ARF (60 mg/Kg), Sty (30 mg/Kg), and San (30 mg/Kg) highest doses have shown ca. 17.20, 24.00, 119.99, 115.89 fold, respectively, improvement in acute paw withdrawal thresholds in non-diabetic mice (Fig. 13). These results showed that FO and ARF have significantly (*P* < 0.05) ameliorated the acute carrageenan-induced inflammatory-pain provoked by carrageenan, as demonstrated previously with analogous compounds [46]. Moreover, Nio-1, Nio-2, Nio-3, Nio-4, Nio-5, and Nio-6 have shown ca. 22.80, 27.98, 25.87, 19.60, 23.99, and 24.80 fold, respectively, improvement in acute paw withdrawal thresholds (Fig. 13). Placebo I and II did not show any significant changes in acute carrageenan-induced inflammatory-pain experiments.

The chronic hind-paw edema method was performed utilizing a modified method described before [35]. The effect of the tested compounds potentials against chronic anti-inflammatory responses was also monitored (Fig. 14). FO (200 mg/Kg), ARF (60 mg/Kg), Sty (30 mg/Kg), and San (30 mg/Kg) highest doses have shown 82.72, 82.90, 72.78, 64.54%, respectively, decrease in hind-paw edema utilizing MRPP plethysmograph (Fig. 14). These results showed that FO and ARF have significantly (*P* < 0.05) ameliorated the chronic hind-paw edema provoked by carrageenan, as demonstrated previously with similar natural phytochemicals [35, 45, 46]. In addition, Nio-1, Nio-2, Nio-3, Nio-4, Nio-5, and Nio-6 have shown 59.09, 89.99, 85.45, 57.27, 78.18, and 75.46%, respectively, decrease in hind-paw edema (Fig. 14). Placebo I and II did not show any significant changes in the chronic hind-paw edema experiments. These results implied that Nio 2 was the most efficient long-term anti-inflammatory formula ameliorating the chronic inflammatory symptoms.

### Anti-inflammatory mechanism of action

To understand the anti-inflammatory mechanism of action attributed to *F. officinalis* and its active phytochemicals, the level of inflammatory-mediators was determined, as done before with similar natural products [47]. FO (200 mg/Kg), ARF (60 mg/Kg), Sty (30 mg/Kg), and San (30 mg/Kg) highest doses, and Nio 1–6 have reduced the levels of cytokines, diminished the levels of the pro-inflammatory cytokines TNF-alpha and IL-6, and elevated the anti-inflammatory factor IL-10 levels (Table 6).

**Table 6:** Determination of the levels of TNF-alpha, IL-6 and IL-10 cytokines

	Vehicle control	FO (200 mg/Kg)	ARF (60 mg/Kg)	Sty (30 mg/Kg)	San (30 mg/Kg)	Nio 1	Nio 2	Nio 3	Nio 4	Nio 5	Nio 6	Placebo I	Placebo II	Ib 100
TNF-alpha	2024.60 ± 102.40	480.90 ± 28.44*	343.90 ± 39.33*	1474.22 ± 10.22*	1388.12 ± 22.11*	1560.40 ± 40.98*	122.55 ± 5.54*	288.53 ± 10.65*	1780.22 ± 40.98*	527.70 ± 33.54*	704.33 ± 37.44*	2036.40 ± 122.60	2029.12 ± 166.87	533.5 ± 96.03
**IL-6**	1212.11 ± 84.00	438.25 ± 32.70*	338.88 ± 28.90*	1426.10 ± 22.50*	1378.44 ± 24.30*	1489.48 ± 39.70*	111.12 ± 8.55*	254.33 ± 16.55*	1370.48 ± 39.66*	607.23 ± 23.70*	798.12 ± 43.60*	1236.33 ± 174.55	1208.99 ± 155.65	91.18 ± 29.36
**IL-10**	1672.24 ± 60.11	3099.34 ± 150.44*	4636.64 ± 366.43*	1805.00 ± 18.56*	1795.33 ± 17.99*	1750.11 ± 11.74*	5022.12 ± 125.33*	4860.22 ± 228.23*	1740.43 ± 13.77*	2055.88 ± 100.43*	1988.88 ± 111.22*	1588.54 ± 204.68	1599.45 ± 258.32	114.05 ± 31.44

The results are expressed as pg/mg of protein and reported as the mean ± SEM. * p < 0.05

## Discussion

Complementary medicine has grown attention in the past years due to their activities in improving human-health effectively, preventing ailments, lower side-effects, and lower costs when compared to conventional treatment [48]. Medicinal herbs are regularly employed to ameliorate different serious disorders like restoring metabolic balance and the management of inflammatory disorders [49]. Thus, phytochemical standardization, fractionation of herbs, isolation, identifying their active compounds responsible for their potentials, and their mechanism of action are crucial for today’s medicine [45]. Moreover, identification and optimization of the pharmacokinetic-characters of herbal drugs are crucial for prediction, monitoring, and improving theirs in vivo biological activities [50]. *Fumaria officinalis* (*F. officinalis*, Papaveraceae) is an annual herb cultivated in Asia and Europe, known for its isoquinoline alkaloids and polyphenols [51]. In Asian folk medicine, the plant is known for its antioxidant properties and used in management of skin disorders, Alzheimer’s disorder, cystitis, rheumatism, and arthritis [52]. Some preliminary experiments have been done exploring *F. officinalis* hypoglycemic activity utilizing in vitro protocols [5]. Nevertheless, this work is the first in-depth account of *Fumaria officinalis* bio-guided phytochemical investigation identifying its main active ingredients, optimizing their pharmacokinetics via various niosomal preparations to enhance their in vivo antineuropathic and anti-inflammatory potentials, and exploring their main mechanism of actions.

The progress in preparing niosomal novel drug delivery systems is a milestone in enhancing the efficacy of various conventional and complementary medicines via improving their pharmacokinetic properties [25].

The in vitro release pattern of the tested niosomes showed that Nio 2 had the fastest release, compared to other formulas (Fig. 4). Nio 2 has shown to have the most superior results related to the normalization of the acute BGL, when compared to FO, ARF, and other formulas. The subchronic and chronic results strengthen the acute findings, that Nio 2 was the most efficient anti-diabetic agent when compared to FO, ARF, and other formulas. These results prove that Nio 2 is efficient in normalization of the BGL not only on short terms, but also on longer terms as seen with similar compounds [24, 29]. Moreover, the insulin secretagogue investigation results prove that Nio 2 is the most efficient formula in their insulin-secretagogue potentials. Furthermore, the diabetic neuropathy results proved that Nio 2 was the most efficient formula combating diabetic hyperalgesia and diabetic mechanical allodynia, as demonstrated before [13, 27]. FO and ARF have shown a dose-dependent alpha-amylase and alpha-glucosidase inhibitory effects (Table 4). Additionally, the antioxidant data proved that Nio 2 is the most efficient in vivo antioxidant formula, and these antioxidant potentials together with their long-term hypoglycemic activities might be responsible for ameliorating diabetic neuropathy symptoms.

The anti-inflammatory results implied that Nio 2 was the most efficient anti-inflammatory formula ameliorating the acute inflammatory pain. Furthermore, the reduction of the pro-inflammatory TNF-alpha and IL-6, elevation the anti-inflammatory factor IL-10 levels, and amelioration of oxidative-stress might be the main mechanism responsible for their anti-inflammatory activities, as previously demonstrated [38, 46].

## Conclusion

The current study strengthens the folk herbal medicine usage of *Fumaria officinalis* in acute and chronic pain, inflammation, and neuropathy. In-vivo bio-guided fractionation and chromatographic phytochemical analysis showed that the alkaloid-rich fraction (ARF) is the most active fraction and that ARF contained two major alkaloids; Stylopine 48.3%, and Sanguinarine 51.6%. In-vitro optimization, analytical, and in vivo biological investigations showed that Nio 2 was the most optimized niosomal formulation. This optimized niosome, Nio 2, worked by improving the pharmacokinetic properties of ARF developing adequate entrapment efficiency, rapid degradation, and acceptable stability in simulated GI conditions. FO, ARF, and Nio 2 are the most potent antidiabetic and anti-inflammatory compounds. Also the reduction of the pro-inflammatory TNF-alpha and IL-6, the elevation the anti-inflammatory factor IL-10 levels, and the amelioration of oxidative-stress might be the main mechanism responsible for their antinociceptive and anti-inflammatory activities. When correlating comparable concentrations, Nio 2 has shown superior efficacy compared to ARF. This study might offer a promising practical oral formulation ameliorating various inflammatory conditions and diabetic complications especially neuropathic-pain, for further research.

## Data Availability

The authors confirm that the data supporting the findings of this study are available within the article.

## Abbreviations

*F. officinalis*: *Fumaria officinalis*
FO: *Fumaria officinalis*
GB: Glibenclamide
ARF: Alkaloid rich fraction
Nio 1–6: Niosome formulations 1–6
BGL: Blood glucose level
Sty: Stylopine
San: Sanguinarine
SEM: Standard error of mean
NC: Normal control
PDI: Poly-dispersity index
GSH: Reduced glutathione level
DC: Vehicle-treated diabetic control
HbA1c: Glycated hemoglobin
SIL: Serum insulin level
PWT: Paw withdrawal threshold
Ib: Ibuprofen
EE: Entrapment efficiency
CAT: Catalase serum level
TNF: Tumor necrosis factor
IL: Interleukin
RP: Reversed phase
ca.: Approximately
LPO: Lipid peroxidase
TBARS: Thiobarbituric acid reactive substances
TRA: Tramadol
